# Use of Atmospheric Budget to Reduce Uncertainty in Estimated Water Availability over South Asia from Different Reanalyses

**DOI:** 10.1038/srep29664

**Published:** 2016-07-08

**Authors:** Dawn Emil Sebastian, Amey Pathak, Subimal Ghosh

**Affiliations:** 1 Department of Civil Engineering, Indian Institute of Technology Bombay, Powai, Mumbai–400 076, India; 2 Interdisciplinary Program in Climate Studies, Indian Institute of Technology Bombay, Powai, Mumbai–400 076, India

## Abstract

Disagreements across different reanalyses over South Asia result into uncertainty in assessment of water availability, which is computed as the difference between Precipitation and Evapotranspiration (P–E). Here, we compute P–E directly from atmospheric budget with divergence of moisture flux for different reanalyses and find improved correlation with observed values of P–E, acquired from station and satellite data. We also find reduced closure terms for water cycle computed with atmospheric budget, analysed over South Asian landmass, when compared to that obtained with individual values of P and E. The P–E value derived with atmospheric budget is more consistent with energy budget, when we use top-of-atmosphere radiation for the same. For analysing water cycle, we use runoff from Global Land Data Assimilation System, and water storage from Gravity Recovery and Climate Experiment. We find improvements in agreements across different reanalyses, in terms of inter-annual cross correlation when atmospheric budget is used to estimate P–E and hence, emphasize to use the same for estimations of water availability in South Asia to reduce uncertainty. Our results on water availability with reduced uncertainty over highly populated monsoon driven South Asia will be useful for water management and agricultural decision making.

Understanding of freshwater availability is of immense importance in assessing socio-economic and environmental impacts of climate and demographic change^1^ and also for climate adaptation to ensure energy and food security^2^. Estimation of available water in South Asian monsoon region is the most important step for agricultural water management considering the growing trend of population in that area and the geographical locations of major river basins such as Indus, Ganga, and Brahmaputra etc. This region is also highly sensitive to climate change and variability^3^. Traditional assessment of available freshwater involves estimation of runoff and/or stream flow^4^,^5^,^6^, which needs rigorous hydrological simulations. The other approach to estimate the approximate water availability is to compute the difference (P–E) between Precipitation (P) and Evapotranspiration (E), which is widely practiced^7^,^8^,^9^,^10^,^11^,^12^,^13^,^14^,^15^ due to its simplicity. However, at a local/watershed level, a water manager does not rely on reanalysis due to its poor performance, but on the observed/gauged data. Understanding of water availability for growing population and its trend at a meso scale (large region such as South Asia) needs the use of reanalysis, due to the poor monitoring network for multiple hydrological variables to a larger spatial extent.

A major challenge in this method of estimation of water availability from different reanalyses is the uncertainty across them in different variables. This has been reported in literature. *Misra et al*.^16^ have shown significant differences in the climatology of evaporation, low-level winds and precipitable water fields over India, where three reanalyses, National Centre for Environmental Prediction ver-2 (NCEP-R2), Climate Forecast System Reanalysis (CFSR) and Modern Era Retrospective-Analysis for Research and Application (MERRA) have been used. They have argued that this difference can be attributed to the uncertainty in the contribution from different oceanic and land sources. Land sources play major role in NCEP-R2, while oceanic sources contribute more in CFSR. *Shah and Mishra*^17^ have evaluated the three high-resolution reanalysis products, MERRA, the Interim ECMWF Re-Analysis (ERA-Interim), and CFSR for assessment of monsoon season drought in India. They have found different biases in the precipitation and temperature estimates from the different reanalysis sets when compared with the observed meteorological data. They have also found that the reanalyses products largely fail to reproduce the characteristics of monsoon season precipitation and temperature over India. Different bias may lead to uncertainty across reanalyses and is one of the main hurdles to use the reanalysis datasets or derived parameters for water management. Inconsistencies between reanalyses were also found by *Annamalai et al*.^18^ in the definition of weak and strong monsoon years based on typical monsoon indices such as All-India Rainfall (AIR) anomalies^19^ and the large-scale wind shear based dynamical monsoon index (DMI)^20^. However, common features such as composite flow patterns associated with weak and strong monsoons and dominant mode of intra-seasonal variability that describes the latitudinal displacement of the tropical convergence zone from its oceanic-to-continental regime were also observed by them. Here we attempt an alternative approach of atmospheric budget to compute P–E over South Asia, which is considered as the proxy to water availability.

*Lorenz and Kunstmann*^21^ found that the atmospheric moisture budget was more balanced compared to the terrestrial budget. The study considered the change in storage in surface budget to be negligible. Studies done by *Trenberth and Guillemot*^22^ concluded that the hydrological cycle components are better represented by atmospheric budget and it was later supported by *Trenberth et al*.^23^ which compared the moisture and energy transport across eight reanalysis data sets. Based on their conclusion that ERA Interim is more reliable in capturing moisture and energy transports, *Trenberth and Fasullo*^24^,^25^ studied the moisture and energy transports over the major land masses using ERA Interim data set. These studies further strengthened the fact that P–E computed from atmospheric moisture budget are more reliable estimates and reproducible than separate estimates of E and P. To further strengthen the hypothesis, they found consistency of water budget estimates with the energy budget. They have argued that the water cycle is integrated to the energy cycle through evaporative cooling at the surface and latent heating of the atmosphere and hence such estimates from atmospheric budget provides a commentary on accuracy of observational estimates. However, such analysis is yet to be applied for South Asian region and here we perform the same for South Asian monsoon region. We further hypothesize that the improved estimates of water availability in terms of P–E compared to individual estimates of P and E result into improvements in consensus across reanalysis. We compare our results with observed station derived gridded and satellite data, specifically for inter-annual variability to see the improvements.

## Results

**Figure 1:**
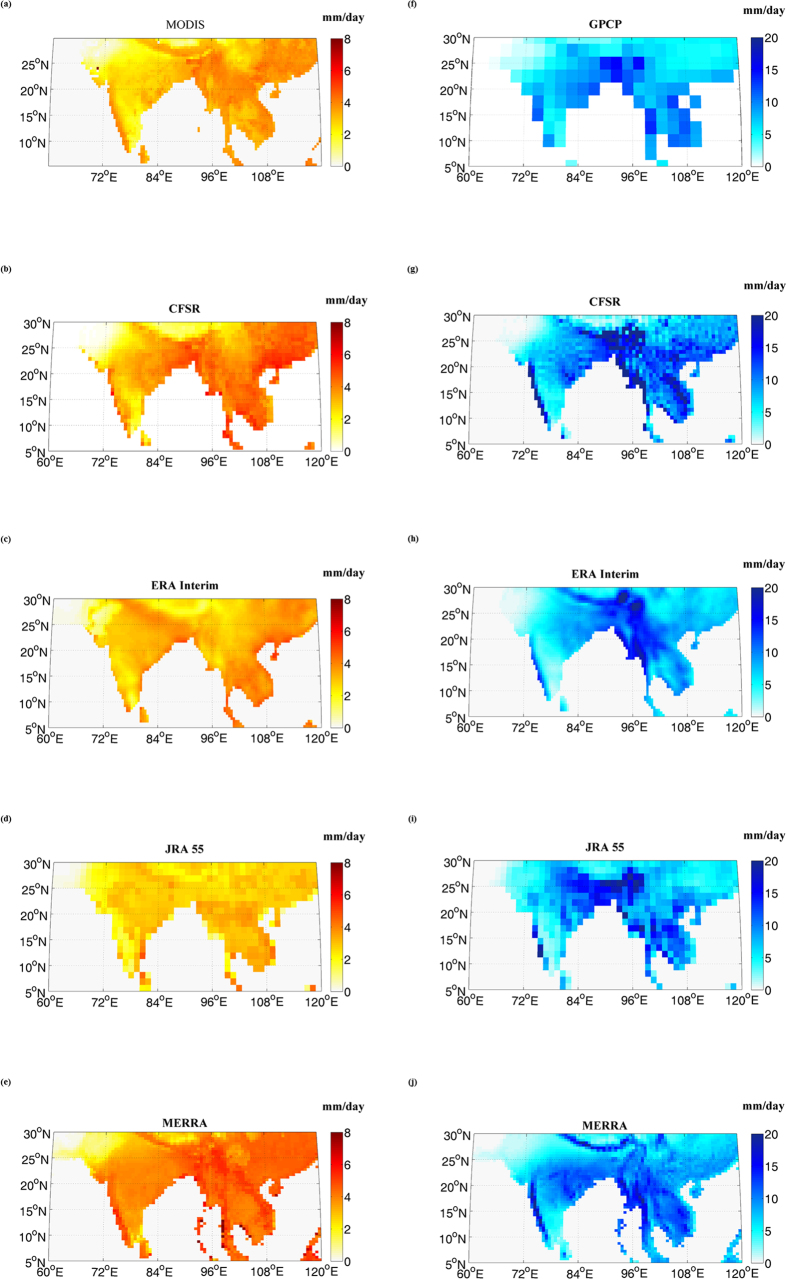
Spatial Variations in (**a–e**) Evapotranspiration and (**f–j**) Precipitation for different data sets; MODIS (**a**), GPCP (**f**), CFSR (**b,g**), ERA-Interim (**c,h**), JRA-55 (**d,i**), and MERRA (**e,j**). The maps are generated with MATLAB 2014a (http://in.mathworks.com/support/sysreq/sv-r2014a/).

**Figure 2:**
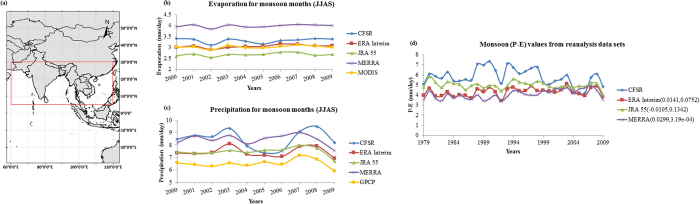
(**a**) The study area, South Asia where the analysis is being performed; (**b,c**) Mean evapotranspiration (E) and Precipitation (P) values, respectively for monsoon months of 2000–2009 period. (**d**) Mean (P–E) values for monsoon months for the period 1979–2009 using direct estimates of precipitation and evaporation as given in reanalyses data sets. The trend values of each time series of P–E are presented as the first terms in the parenthesis within the legend and the second terms denote their significance. The analysis is performed only over the land region. The map is generated with GrADS (http://www.iges.org/grads/).

We use four high resolution reanalysis data, MERRA, CFSR, ERA‒Interim and JRA‒55 for the computation of the water availability in terms of P–E over monsoon dominated highly populated South Asian landmass [5^0^N to 30^0^N and 60^0^E to 120^0^E]. The reanalyses products have varying ability to resolve topography and coastlines due to the differences in spatial resolutions. Here we consider the reanalyses data which are of comparable spatial resolutions and have avoided the use of coarse resolution reanalysis, such as NCEP/NCAR or ERA‒40. MERRA reanalysis data^26^ was developed by the NASA’s Global Modelling and Assimilation Office with an aim to improve the hydrological cycle and employ 3DVAR data assimilation scheme. They provide data at a resolution of 1/2° × 2/3° from 1979 to present at 6 hourly, daily and monthly timescales at 72 pressure levels. CFSR data set^27^ developed by NOAA/NCEP aimed at improving the short comings of its widely used predecessor NCEP-NCAR reanalysis set and employ 3DVAR assimilation scheme like the MERRA data. CFSR data are available at hourly, 6 hourly and monthly time scales from 1979 to 2009 at T382 horizontal resolution over 64 pressure levels. ECMWF developed the ERA‒Interim data set^28^ to replace ERA‒40, employing the 4DVAR assimilation scheme generating data at 60 pressure levels. They provide 6 hourly, daily and monthly data from 1979 to present at T255 resolution. The JRA‒55 reanalysis data set^29^, developed by JMA is a 55 years reanalysis dataset, extending back to 1958 taking into account the deficiencies faced by their previous 25 year JRA‒25. It employs 4DVAR with Variational Bias Correction (VarBC) for its data assimilation and provide data at TL319 horizontal resolution and 60 pressure levels. Long term variation of monsoon water availability was studied for the years from 1979 to 2009. We first compare the precipitation and evaporation values from the different reanalyses with the GPCP and MODIS data respectively (Fig. 1). Here, we observe that the E from ERA‒Interim and MODIS are comparable, whereas considerable overestimation is observed in MERRA reanalysis. Figure 1(f–j) show overestimation of monsoon precipitation by all the reanalyses. Considering the varying resolution across the reanalyses and also in observed satellite data, we also compare the spatial average of P and E over the land region for the monsoon months. We present the results for the summer monsoon period June, July, August and September (JJAS), during which the rainfall covers almost 80% of the annual rainfall^30^,^31^,^32^. We find that MERRA is overestimating both E and P; whereas, ERA‒Interim remains closer to the observed data for both (Fig. 2). Figure 2(d) shows the uncertainty across different reanalyses in terms of the estimated P–E. Cancellation of positive biases, while subtracting E from P, makes the P–E from MERRA comparable to ERA‒Interim; however considerable uncertainty exist across reanalyses with higher estimation of P–E by CFSR and JRA‒55. This is due to overestimation of precipitation by CFSR and underestimation of evaporation by JRA‒55. We find that the differences in precipitation as estimated from multiple reanalysis can be attributed to varying assimilation scheme. ERA‒Interim and JRA‒55 uses 4DVAR assimilation scheme, whereas MERRA and CFSR uses the 3DVAR scheme. 4DVAR assimilation scheme considers the dynamics and physics of the forecast model during assimilation and it can assimilate all observational data including those that are derived from model variables, like the precipitation. This could be the reason for the better performance of ERA‒Interim and JRA‒55 in simulating precipitation, employing 4DVAR assimilation scheme. ERA- Interim and MERRA have positive trend, whereas, CFSR has negative trend in P–E, which are significant at 10% significance level. JRA‒55, however does not show any statistically significant trend.

**Figure 3:**
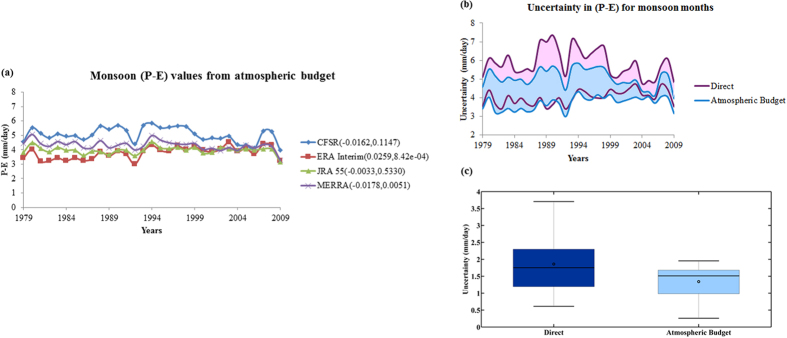
(**a**) Mean (P–E) values for monsoon months with different reanalysis for the period 1979–2009 derived from atmospheric moisture budget. The trends are presented in a similar way as Fig. 2(d). Uncertainty in P–E values estimated as the difference between maximum and minimum values represented as bands (**b**), box plot (**c**).

We use the divergence of moisture flux to directly compute the monthly P–E from Eq. (1) [details in Methods] in an attempt to reduce uncertainty across reanalysis in computing water availability. First we plot the convergence term of Eq. (1) [same as P-E] for the monsoon months (JJAS) with the wind profile [Supplementary Fig. S1]. The convergence computed from CFSR shows prominent orographic impacts at the Western coast of India (the Western Ghats region) and at the North-East India, which are not as distinct for MERRA, JRA‒55 and ERA‒Interim. The wind profile remains almost similar across reanalyses and hence the differences can be attributed to the amount of moisture transported from ocean to land. Furthermore, the computation of divergence often gives error in the coastal and mountainous regions at monthly or coarser temporal scales^33^. The convergence values computed for June, July, August and September are plotted in Supplementary Figs S2–S5 for CFSR, ERA-interim, JRA 55 and MERRA, respectively. For CFSR, the month to month variability within monsoon season does not really show the distinct observed intra-seasonal patterns of P–E. CFSR shows almost similar values of divergence for all the 3 months of June, July and August over the core monsoon zone, the Central India. MERRA, JRA 55 and ERA-interim capture the observed pattern of convergence with peak values during July and August over Central India. The time series of seasonal monsoon P–E values obtained from different reanalyses are presented in Fig. 3(a). The agreement among reanalyses have significantly improved with atmospheric budget, when compared with the P–E values obtained from individual components (P and E separately). This may be attributed to the fact that while computing P–E from atmospheric budget, only Type A and Type B reanalysis variables are employed which are more dependent on the observations^34^. The variables like precipitation, evaporation and runoff are of *Type*–*C* category in reanalysis and hence are completely model dependent. This may lead to less reliable estimates and inconsistency across the different reanalysis datasets due to varying models. Here, we find that the consistency in water availability estimates could be improved using atmospheric quantities (*Type–A* and *Type–B* variables) which are better captured by the reanalysis. To further clarify this statement, we have now plotted the uncertainty band for P–E computed directly and with atmospheric budget (Fig. 3(b)). The width of band for the later one is lower and hence we conclude that the estimation with atmospheric budget reduces the uncertainty in water availability. Here, the uncertainty is quantified for both the estimates of P–E during each of the year in terms of the differences between the maximum and the minimum from multiple reanalyses. These uncertainties are presented with box plots (Fig. 3(c)), where the width of the box presents year to year variability. The figure shows considerable reduction of uncertainty with atmospheric budget. The MERRA and ERA‒Interim sets give higher values for P–E when computed from atmospheric budget compared to those obtained by using direct estimates of precipitation and evaporation. We find that the estimates of P-E are getting reduced in case of CFSR and JRA‒55, when atmospheric budget is used.

We further test the hypothesis that such increase in agreement across the three reanalyses can be attributed to the individual improvements in P–E values after the application of atmospheric budget. The improvements are quantified in terms of reduction of closure term of water cycle [Eq. (2), Details in Methods]. To compute the magnitude of the closure term over South Asia, we use the satellite based water storage data from Gravity Recovery and Climate Experiment (GRACE), available at http://grace.jpl.nasa.gov^35^,^36^,^37^. GRACE has major limitations in terms of resolving terrestrial features such as topography and hence they are not suitable for fine resolution local/regional studies. When using GRACE data in regions with fine coastal detail, ocean signals can bleed into land areas and it can be problematic in the computation of terrestrial budget. The change in storage from GRACE is therefore, compared with that of MERRA Land and GLDAS (Supplementary Fig. S6). The values obtained from both GLDAS and MERRA Land are comparable (Supplementary Fig. S6), whereas the GRACE provides slightly higher values of storage in the monsoon months. This may be attributed to the fact that both MERRA Land and GLDAS do not consider the effect of irrigation, which has a significant impact on the hydrological parameters including storage^38^,^39^,^40^. GRACE, being a satellite based observed data, is more reliable than model simulated outputs such as GLDAS. Here we perform the analysis for a larger region, i.e., South Asia, without focussing on spatial pattern and variability at a fine resolution, affected by topographical features. GRACE data have been used across different parts of the globe for such large scale studies of ground water storage^41^,^42^. Application of GRACE in understanding water storage for Indian subcontinent may also be found in *Rodell et al*.^42^ and *Panda et al*.^43^. Due to the non-availability of the runoff data over such a large region of South Asia, we use the output from Global Land Data Assimilation System (GLDAS) from http://disc.sci.gsfc.nasa.gov/services/grads-gds/gldas^44^,^45^. The land surface models (LSMs) used in GLDAS are National Centre for Environmental Prediction/Oregon State University/Air Force/Hydrologic Research Lab (NOAH)^46^,^47^, MOSAIC^48^,Community Land Model (CLM)^49^ and Variable Infiltration Capacity (VIC)^50^,^51^ Models. *Zaitchik et al*.^42^ evaluated the river discharge of world’s selected rivers from gridded GLDAS data using a source to sink (STS) routing scheme. Simulated river discharge from the four LSMs of GLDAS, for Ganges and Mekong, the two major rivers in the current study area exhibited a correlation higher than 0.8 with the historical gauge data from Global Runoff Data Centre (GRDC) in majority of the LSMs. All the four LSMs underestimated the discharge for Ganges, however CLM was most efficient as it simulated the runoff which has less than 20% bias^42^. The discharge values for Mekong River provided by the GRDC was higher than those estimated by *Dai and Trenberth*^49^ and *Dai et al*.^50^. We find that the GLDAS-STS simulated values agreed more with the latter. The study however, concluded that there was significant differences in the simulated runoff from the different LSMs, even when the meteorological forcing data were the same. Therefore, in the present work, we have considered four LSMs and the spread across these LSMs represent the uncertainty in the closure terms attributed to the use of multiple LSMs^52^,^53^.

**Figure 4:**
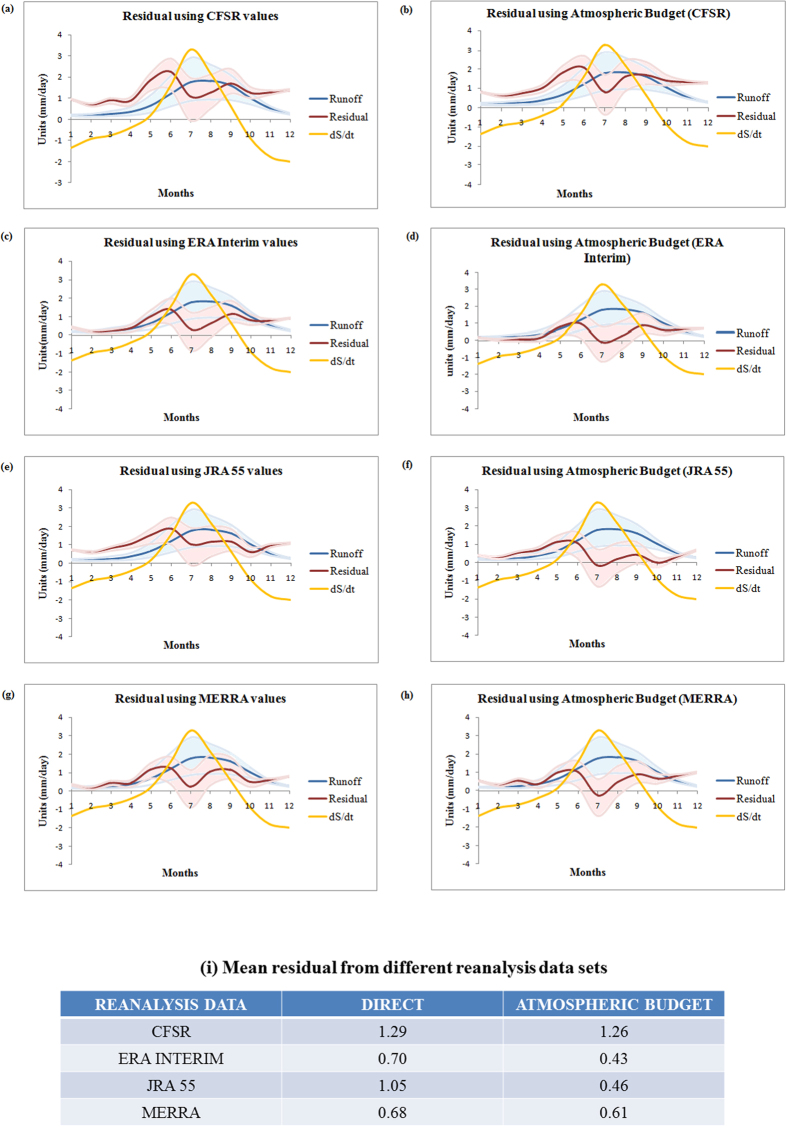
The climatology of runoff from GLDAS (with uncertainty band resulting from the use of multiple LSM), storage change (from GRACE) and the residual (with uncertainty band) obtained using P–E estimates from CFSR (**a**,**b**), ERA Interim (**c**,**d**), JRA 55 (**e**,**f**) and MERRA (**g**,**h**) over South Asia. The mean residual values are presented in (**i**).

The climatology of runoff (with uncertainty band resulting from the use of multiple LSM), storage change (from GRACE) and the residual (with uncertainty band) are presented in Fig. 4. We find that the ensemble mean residuals have been improved for all the reanalyses (Fig. 4(i)). The improvement is the maximum for JRA‒55, followed by ERA‒Interim. A similar analysis was done by *Trenberth and Fasullo*^25^ over the entire Eurasia and there are significant difference in the climatology of various hydrological variables, especially runoff and soil moisture from the present study, which can be attributed to the larger domain (Eurasia) not entirely affected by monsoon. The mean residuals obtained from both, individual components and atmospheric budget are similar for MERRA. A better evaluation should be performed with observed runoff data rather than the GLDAS model outputs, as the estimated uncertainty (width of bands) in Fig. 4 are of similar magnitude to the ensemble mean values of residuals. This is one of the limitations of the evaluation procedure used.

We also evaluate if the agreement across reanalyses, observed in terms of biases, are also reflected in inter-annual variability. We measure the agreement in inter-annual variability in terms of correlation across reanalyses. We find negative correlation between CFSR and MERRA when P–E are computed from individual components (Table 1). This makes the computed water availability over South Asia not reliable and highly uncertain. When we apply atmospheric budget, we find the improvement in the above mentioned correlation from −0.18 to 0.68. Improvements in correlation are also observed for the other pairs. However the correlation coefficients of MERRA and CFSR with ERA‒Interim are reduced. For further evaluation, we compute the P–E from observed gridded precipitation and satellite product of E. The observed precipitation data is obtained from the Global Precipitation Climatology Project (GPCP) monthly precipitation data provided by the NOAA/OAR/ESRL PSD, Boulder, Colorado, USA, from their Web site at http://www.esrl.noaa.gov/psd/which combines both observations and satellite precipitation data into 2.5° × 2.5° grids^54^. We use the satellite data for E, which is taken from Moderate Resolution Imaging Spectroradiometer (MODIS)^55^. Here we perform our entire analysis over the land mass region of South Asia. We find significant improvements in the correlation between the observed/satellite derived P–E values and those from MERRA, CFSR and ERA‒Interim when it is derived from atmospheric budget. Improvements are not observed for JRA‒55. Overall, based on the improved agreement across reanalyses, as well with observed/satellite derived data, it is recommended to use atmospheric budget to compute P–E for analyzing water availability rather than the use of individual components of P and E from different reanalyses.

To understand the consistency of P–E, derived from atmospheric budget, with the energy budget, we compare it with the divergence of latent energy flux, which is the energy flow due to the moisture exchange processes. We perform this consistency check only for the ERA‒Interim reanalysis data, considering the availability of required data (Supplementary Figs S7 and S8). Following *Trenberth and Fasullo*^24^,^25^, we include top-of-atmosphere (TOA) radiation from the Clouds and the Earth’s Radiant Energy System (CERES) observations, atmospheric energy quantities and transports from reanalyses for energy cycle. The annual mean P–E from ERA‒Interim using separate estimates of precipitation and evaporation was found to be 1.54 mm/day and that from atmospheric budget was 1.26 mm/day. This when multiplied with a factor of −29.1 (considering appropriate unit conversions to the latent heat of vaporization of water as mentioned by NCAR Community Climate Model 2 as 2.5104 × 10^6^ Jkg^−1^), gives divergence of latent energy flux as −44.8 Wm^−2^ and −36.7 Wm^−2^ respectively, compared to −38.0 Wm^−2^, as provided by the ERA Interim, thus supporting the argument that better estimates of water availability can be derived from atmospheric budget (Supplementary Fig. S7(d)).

We further study the Water budget in a CMIP5 coupled ESM for South Asia. We use the outputs from MIROC ESM and the results are presented in Supplementary Fig. S9. The figure shows that the P–E computed with both direct method and atmospheric budget are comparable. However, we do not observe a comparable Runoff (R) value to make P–E-R = 0, which is expected over the land region as per the assumption of fully coupled models. Further investigation shows that the ESM under-simulates R during monsoon and over-simulates the same during non-monsoon months. This probably results to a positive value of P–E-R during monsoon months over South-Asia. Such seasonal discrepancy between P–E and R leads to a very challenging research problem, and the hydrologic analysis required will be non-trivial. We consider this as a potential research area for future.

## Conclusion

Our results show the improvements obtained in the estimated water availability from different reanalyses, when computed with atmospheric budget rather than individual reanalyses values of E and P. The following conclusions are derived from the present work:

1. There is huge uncertainty in the estimates of P–E, when computed from different reanalysis and a significant part of this uncertainty comes from the deviation of CFSR variables from majority of reanalysis.
2. Use of atmospheric budget improves the estimates of P–E as compared to individual estimates. These improvements are visible in terms of closure term of water cycle over South Asia, the maximum improvement was seen for JRA‒55.
3. Use of atmospheric budget improves the correlations between the estimates of seasonal P–E obtained from different reanalysis, i.e., results into improvements in the agreement across reanalysis for inter-annual variability.
4. Use of atmospheric budget results into improved correlation between P–E obtained from reanalysis and gridded/satellite based observed data. Atmospheric budget derived E-P also shows improved consistency with the energy budget over South Asia.

Few limitations of the present study are:

1. *Reliability of GLDAS and GRACE data*: GRACE data due to its coarser resolution is not capable of resolving the various topographic effects and coastal line. GLDAS do not consider human intervention and hence this may not always present the real hydrological situation.
2. *Closure term as measure of reliability of water availability estimates:* Considering the magnitude of closure term as a measure of reliability has limitation as the tools used in reanalyses or GRACE do not explicitly consider water conservation.

To minimize the impacts of these limitations, we perform the analysis over a larger spatial region (for issues associated with the resolution of GRACE) and present the uncertainty associated with multiple models from GLDAS.

Our results suggest the regional planners need to be aware of the uncertainty across different reanalyses before using them for water management. It is also recommended to use atmospheric budget for computation of water availability in terms of P–E rather than based on individual values of P and E. Considerable differences in P–E computed with atmospheric budget and individual components probably indicate the possible limitations of models in simulating P and E separately. This probably attributes to inadequate representation of cloud and convective scheme for P and land surface schemes for E. Follow on research activities should include a better representation of above mentioned schemes targeted to reduce the closure term as well the uncertainty for improved water resources decision making.

## Methods

We first apply the atmospheric moisture budget equation to understand the atmospheric storage and transport of moisture over the South Asian region, 5^0^N to 30^0^N and 60^0^E to 120^0^E (Fig. 2(a)). The equation is given by^22^,^56^:


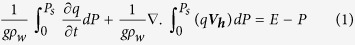


where, q is the specific humidity, **V**_***h***_ is the wind velocity vector, both of which are measured at different pressure levels, starting from surface pressure P_s_. E and P represent the evaporation and precipitation rates respectively^22^. The first term in the left hand side provides the change in storage, whereas the second term denotes the divergence of moisture flux. For a timescale of monthly or higher order, change in storage of atmospheric moisture is negligible and the E-P value may be equated to the divergence of moisture flux. Here, we follow the same to compute the values of E-P using atmospheric budget. The vertically integrated quantities provided by NCAR which incorporates mass corrections are used in the current study (http://www.cgd.ucar.edu/cas/catalog/reanalysis/).

Following the methodology suggested by Trenberth and Fasullo^24^,^25^, we compute the closure term of water cycle. The closure term of the residual error (Res) is given by:


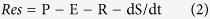


where, R is the runoff and dS/dt is the temporal change in the water storage. Due to non-availability of runoff R, we use the outputs from GLDAS models, NOAH, MOSAIC and CLM. CLM applies finite difference spatial discretisation methods and a fully implicit time integration scheme for the computation of governing equations. It is the land model for NCAR’s coupled Community Climate System Model (CCSM). Mosaic Model is a well-established land surface model and was the first to treat sub-grid scale variability by dividing each grid into a mosaic of tiles. NOAH employs finite difference spatial discretisation methods and Crank Nicholson time integration scheme to numerically integrate the governing equations. VIC mainly focuses on the runoff as represented by the variable infiltration capacity curve.

## Additional Information

**How to cite this article**: Sebastian, D. E. *et al*. Use of Atmospheric Budget to Reduce Uncertainty in Estimated Water Availability over South Asia from Different Reanalyses. *Sci. Rep.* **6**, 29664; doi: 10.1038/srep29664 (2016).

## Supplementary Material

Supplementary Information

## Figures and Tables

**Table 1:** Correlation coefficients between P–E obtained from different reanalysis along with their individual correlation with observed data.

	CFSR	ERA Interim	JRA 55	MERRA	GPCP-MODIS
**(a) Values from Reanalysis Data Sets**					
**CFSR**	1	0.56	0.42	−0.18	0.36
**ERA Interim**	0.56	1	0.47	0.57	0.58
**JRA 55**	0.42	0.47	1	0.39	0.93
**MERRA**	−0.18	0.57	0.39	1	0.75
**(b) Values from Atmospheric Moisture Budget**					
**CFSR**	1	0.42	0.56	0.68	0.75
**ERA Interim**	0.42	1	0.58	0.30	0.80
**JRA 55**	0.56	0.58	1	0.80	0.71
**MERRA**	0.68	0.30	0.80	1	0.89
