# Supplementary material for: Use of Atmospheric Budget to Reduce Uncertainty in Estimated Water Availability over South Asia from Different Reanalyses
Source: Sci Rep. 2016 Jul 8;6:29664. doi: 10.1038/srep29664 (PMC4937427; doi:10.1038/srep29664)
Supplement: Supplementary Information [file srep29664-s1.pdf]

Supplementary Information

for

Use of Atmospheric Budget to Reduce Uncertainty in Estimated Water Availability over South Asia from Different Reanalyses

Dawn Emil Sebastian[1], Amey Pathak[1], Subimal Ghosh[1,2,*]

[1]Department of Civil Engineering, Indian Institute of Technology Bombay, Powai, Mumbai – 400 076, India

[2]Interdisciplinary Program in Climate Studies, Indian Institute of Technology Bombay, Powai, Mumbai – 400 076, India

[*] Department of Civil Engineering, Indian Institute of Technology Bombay, Powai, Mumbai – 400 076, India, Email: subimal@civil.iitb.ac.in; Phone: +91 22 2576 7319; Fax: +91 22 2576 7302

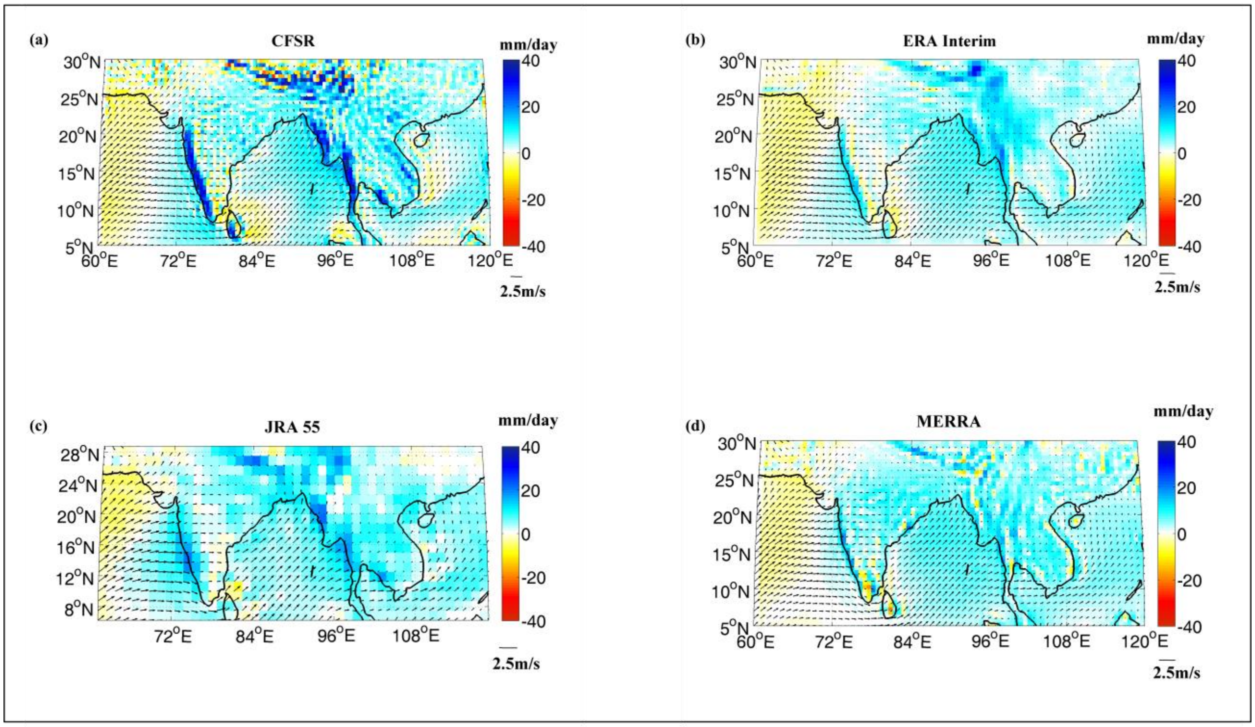

Figure S1: Mean spatial distribution of P-E derived from atmospheric budget for the monsoon (JJAS) months using (a) CFSR, (b) ERA-Interim, (c) JRA-55, and (d) MERRA reanalyses. The maps are generated with MATLAB 2014a (http://in.mathworks.com/support/sysreq/sv-r2014a/)

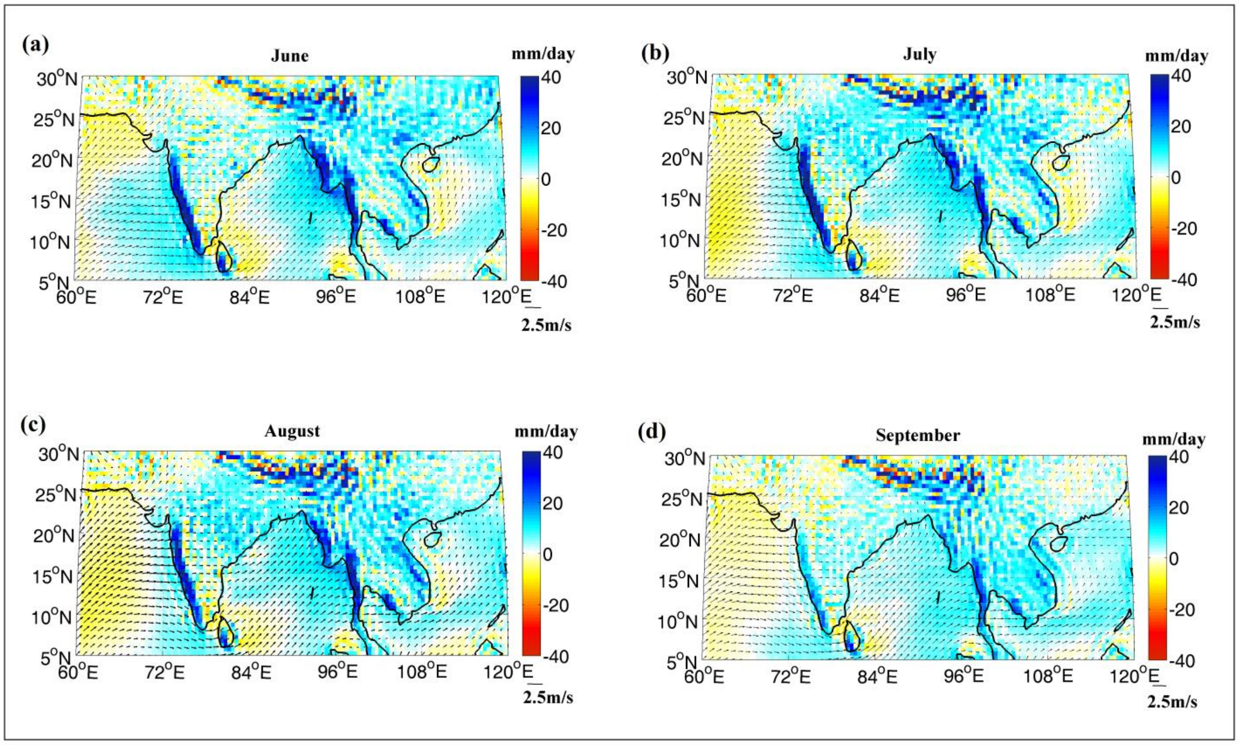

Figure S2: Mean spatial distribution of P-E derived from atmospheric budget for the four monsoon (JJAS) months using CFSR data. The maps are generated with MATLAB 2014a (http://in.mathworks.com/support/sysreq/sv-r2014a/)

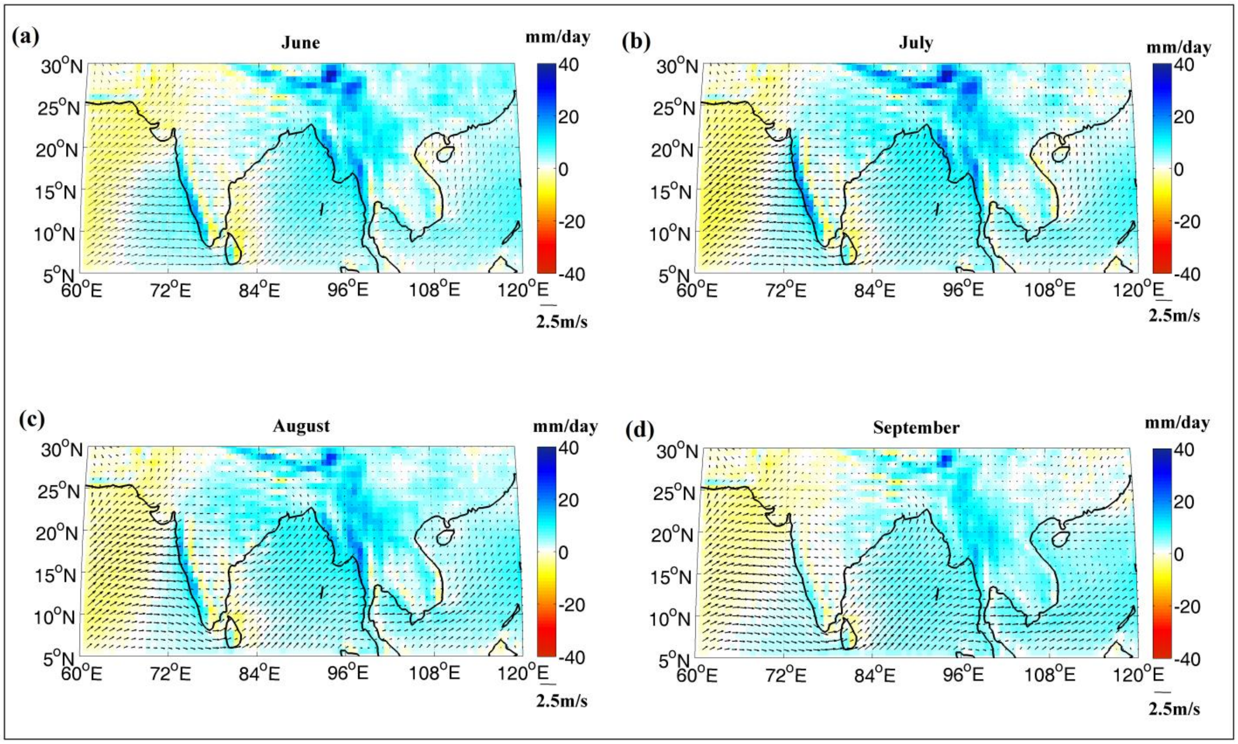

Figure S3: Mean spatial distribution of P-E derived from atmospheric budget for the four monsoon months using ERA Interim data. The maps are generated with MATLAB 2014a (http://in.mathworks.com/support/sysreq/sv-r2014a/)

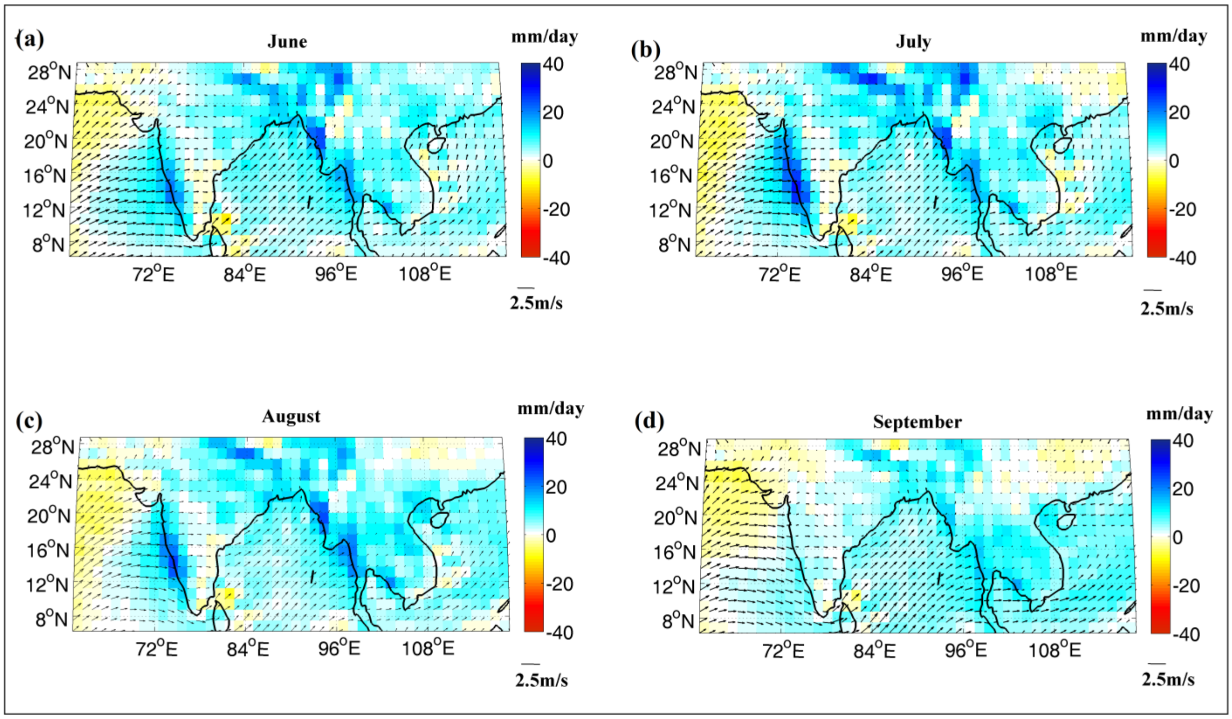

Figure S4: Mean spatial distribution of P-E derived from atmospheric budget for the four monsoon months using JRA-55 data. The maps are generated with MATLAB 2014a (http://in.mathworks.com/support/sysreq/sv-r2014a/)

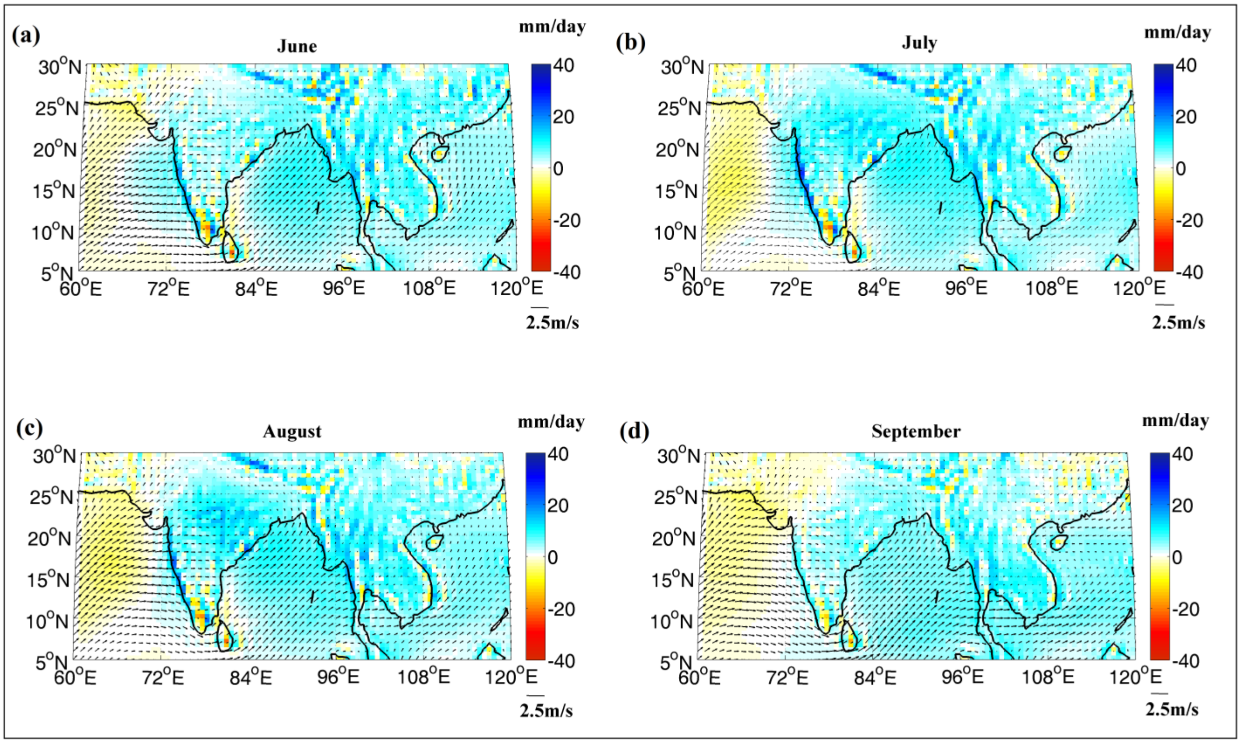

Figure S5: Mean spatial distribution of P-E derived from atmospheric budget for the four monsoon months using MERRA data. The maps are generated with MATLAB 2014a (http://in.mathworks.com/support/sysreq/sv-r2014a/)

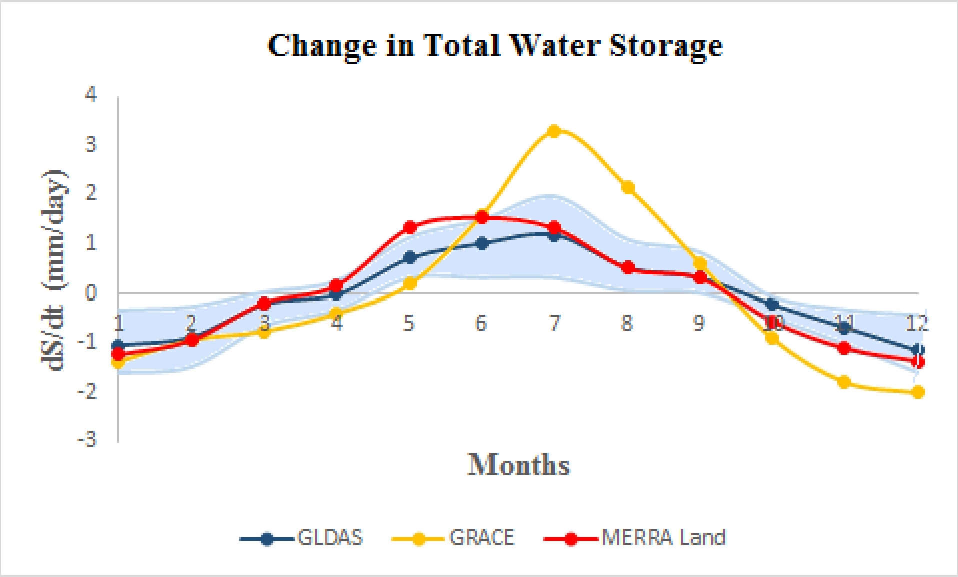

Figure S6: Climatology of change in total water storage over the period 2003 to 2009, when obtained using GRACE, MERRA Land and GLDAS data. The uncertainty in GLDAS value is the difference between the maximum and minimum values as obtained from the different LSMs in GLDAS.

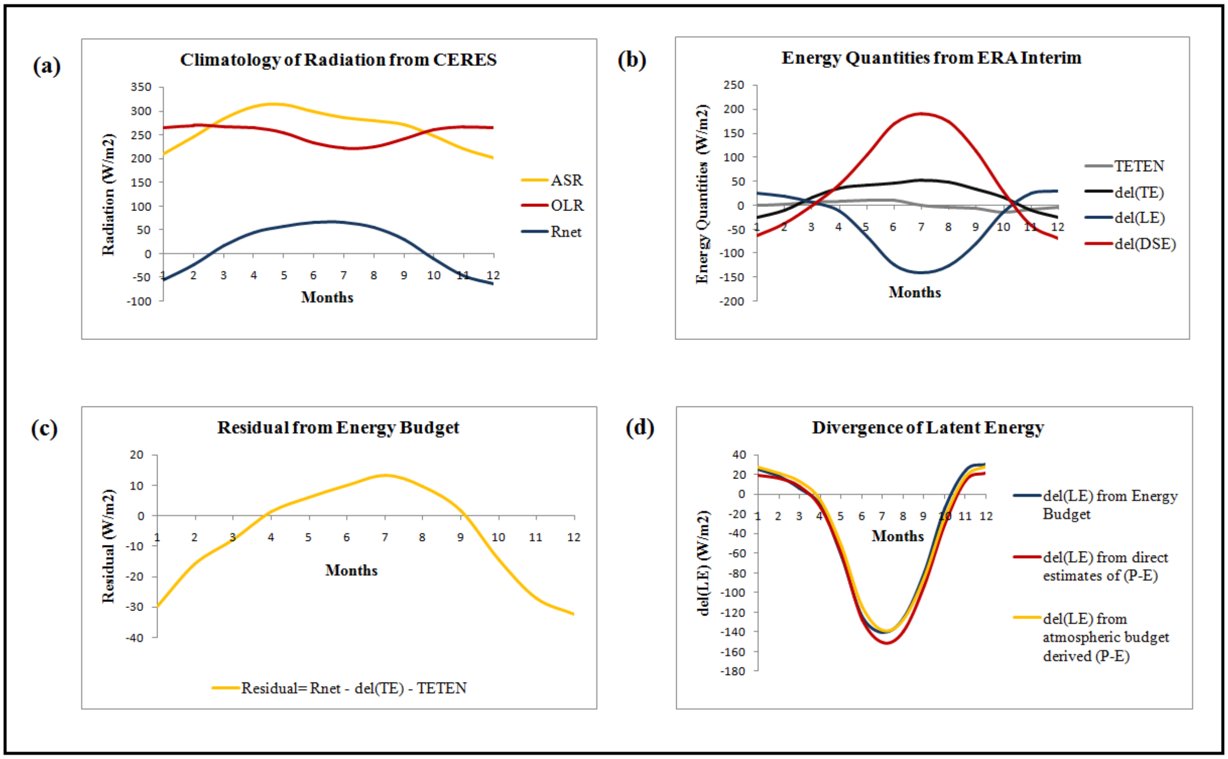

Figure S7: (a) Climatology of TOA radiation as obtained from CERES satellite data showing variability of Absorbed Solar Radiation (ASR), Outgoing Longwave Radiation (OLR) and Net Radiation (Rnet) (b) Atmospheric energy quantities as obtained from ERA Interim showing climatology of divergence of total energy (del(TE)), divergence of dry static energy (del(DSE)), divergence of latent energy (del(LE)) and tendency of total energy (TETEN). (c) Climatology of residual calculated from energy budget derived as: residual=Rnet-del(TE)-TETEN (d) Climatology of divergence of latent energy

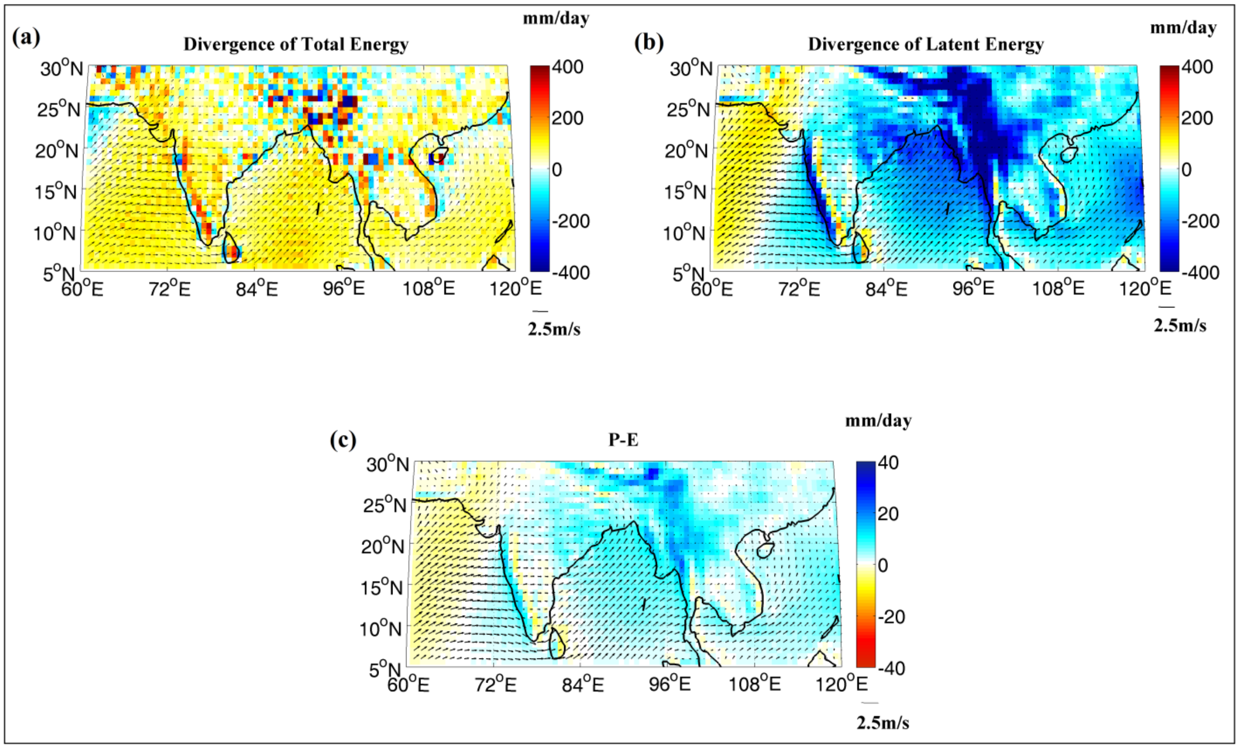

Figure S8: Mean spatial distribution of (a) divergence of total energy (b) divergence of latent energy and (c) (P-E) derived from atmospheric budget over the study region for monsoon months from ERA Interim data set. The maps are generated with MATLAB 2014a (http://in.mathworks.com/support/sysreq/sv-r2014a/)

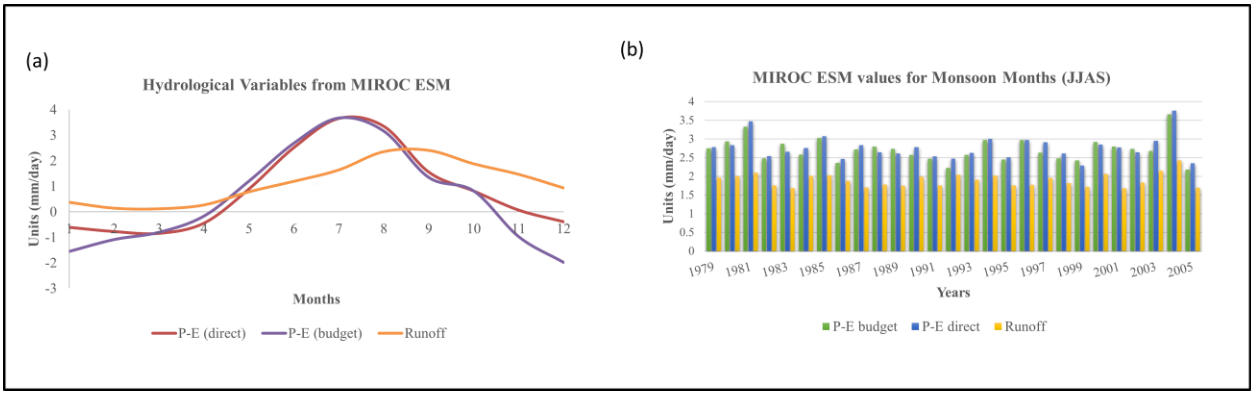

Figure S9: (a) Climatology of hydrological variables as obtained from MIROC-ESM. (b) Spatial mean of hydrological variables for the monsoon months for the same.
